# Maternal vaccination protects dams and prevents *in utero* transmission of Rift Valley fever virus in rats

**DOI:** 10.1128/mbio.00253-26

**Published:** 2026-06-22

**Authors:** Austin T. Hertel, Cynthia M. McMillen, Ryan M. Hoehl, Zachary D. Frey, Anita K. McElroy, Amy L. Hartman

**Affiliations:** 1 Center for Vaccine Research, University of Pittsburgh 6614, Pittsburgh, Pennsylvania, USA; 2 Department of Infectious Disease and Microbiology, School of Public Health, University of Pittsburgh 51303, Pittsburgh, Pennsylvania, USA; 3 Division of Pediatric Infectious Disease, Department of Pediatrics, School of Medicine, University of Pittsburgh 12317, Pittsburgh, Pennsylvania, USA; University of Pennsylvania, Philadelphia, Pennsylvania, USA

**Keywords:** Rift Valley fever, bunyavirus, RNA virus, pregnancy, vaccine, vertical transmission

## Abstract

*In utero* transmission and spontaneous fetal death are hallmarks of Rift Valley fever virus (RVFV) infection in pregnant animals. Compounding evidence indicates pregnant individuals are particularly vulnerable to RVFV, as *in utero* transmission and increased rates of miscarriage have been reported in people infected during pregnancy. Further, human placenta explants are permissive to RVFV infection. Viable vaccine candidates intended for veterinary or human use must protect vulnerable populations, including pregnant individuals and fetuses. Using a pregnant rat model, we show that maternal vaccination with a live-attenuated RVFV lacking the non-structural proteins NSs and NSm (RVFV-delNSs/NSm) is immunogenic, safe, and protective. Dams vaccinated either prior to or during pregnancy were protected from virulent challenge during pregnancy, and we found no evidence of infectious virus in the placentas and fetuses of challenged animals. These studies offer important pre-clinical data in a tractable pregnancy model and serve as a blueprint for evaluating future vaccine approaches designed to protect pregnant individuals and their fetuses.

**IMPORTANCE:**

Rift Valley fever virus (RVFV) poses a formidable threat to pregnant animals and potentially to pregnant individuals and their developing fetuses. Despite this risk, pregnant individuals are rarely included in vaccine trials, leaving this potentially vulnerable population unprotected. Using a live-attenuated RVFV vaccine, we demonstrate that maternal vaccination is safe and protects both pregnant rats and their fetuses from congenital Rift Valley fever. Because live-attenuated vaccines are often contraindicated during pregnancy due to theoretical safety concerns, we further show that vaccination prior to pregnancy provides equivalent protection throughout future pregnancy. Taken together, these findings provide a blueprint for the preclinical evaluation of RVFV vaccines during pregnancy.

## INTRODUCTION

The World Health Organization (WHO) considers the mosquito-borne Rift Valley fever virus (RVFV) a priority for research and vaccine development (1). Although RVFV is endemic to Africa and parts of the Middle East, its mosquito vectors are distributed globally (2–4), posing a risk of emergence in new regions (5). RVFV primarily affects domesticated livestock species (sheep, goats, and cattle) with epizootics marked by waves of spontaneous fetal loss in pregnant animals (6, 7). Occasionally, these epizootics result in human outbreaks in affected regions (8–10). During human circulation, *in utero* transmission of RVFV in humans has been reported, with infants born at full-term with severe hepatic disease (11, 12). Further, a cross-sectional study in Sudan found that pregnant individuals infected with RVFV experienced a fourfold increase in second- and third-trimester fetal loss (13). Collectively, these findings underscore the serious but often overlooked threat RVFV may pose to pregnant individuals and their developing fetuses.

There are currently no licensed vaccines available for human use. Live-attenuated vaccines (LAVs) like Smithburn and Clone-13 are currently used in endemic regions to control the spread of RVFV in livestock (14). However, a key issue with these LAVs is that they retain partial virulence in pregnant animals, causing fetal death and congenital defects (15, 16). Therefore, these vaccines are contraindicated in pregnant livestock and are not suitable for human use. MP-12, another RVFV LAV developed in the 1980s, has been tested extensively in animal models and is protective against lethal RVF disease after a single immunization (17–21). However, there are issues regarding the safety profile of high doses of MP-12 in pregnant animals (22).

To address these concerns, a next-generation RVFV LAV (RVFV-delNSs/NSm) was developed. RVFV-delNSs/NSm was generated by reverse genetics using the parental ZH501 strain that completely lacks the two known RVFV virulence genes encoding non-structural proteins, NSs and NSm (23). Vaccination with RVFV-delNSs/NSm is generally avirulent and provides near-complete protection against wild-type (WT) virus challenge in a variety of species (rodents, sheep, and non-human primates), including pregnant sheep (23–26). A recent study demonstrated that even low doses (20 TCID_50_) of RVFV-delNSs/NSm protect mice from lethal hepatic and encephalitic manifestations of RVF (27). The goal of this study is to evaluate the safety and immunogenicity of RVFV-delNSs/NSm in early-gestation pregnant rats. Compared to sheep, pregnant Sprague-Dawley rats are a more tractable model for the study of *in utero* transmission of RVFV (28), and we have demonstrated their usefulness for testing candidate RVFV vaccines and therapeutics (29, 30). DDVax, a patented human vaccine candidate based on the genetic deletion of NSs and NSm, has advanced toward clinical trials funded by the Coalition for Epidemic Preparedness Innovations (CEPI) (31). Given the conflicting safety data for other RVFV LAVs and the likely exclusion of pregnant individuals from early-stage clinical trials (32), this study provides pre-clinical data to potentially guide vaccination strategies needed to combat the significant risk RVFV poses during pregnancy.

## RESULTS

### Pregnant and non-pregnant rats respond similarly to vaccination with live-attenuated RVFV

**Fig 1:**
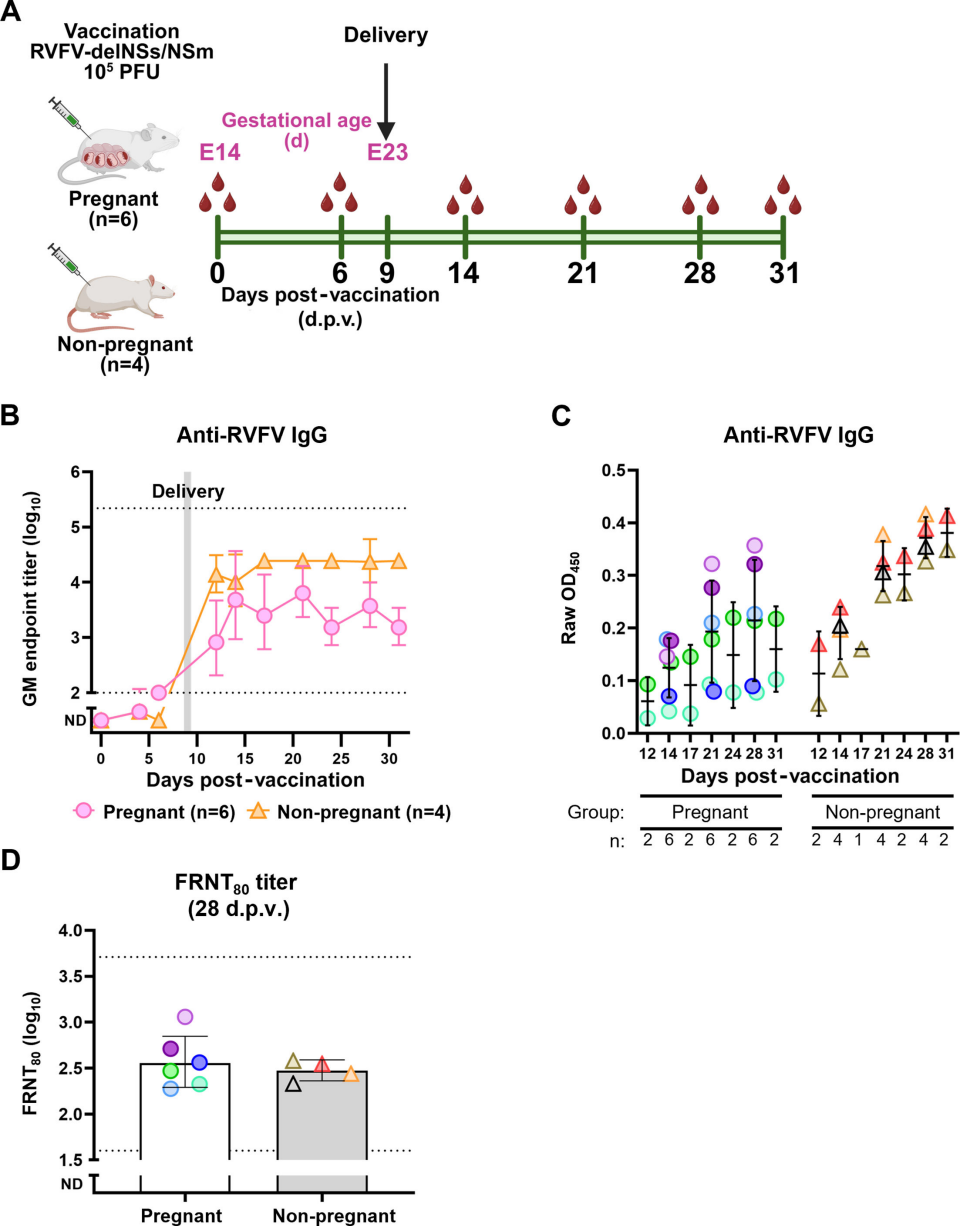
Anti-RVFV IgG and neutralizing antibody responses in pregnant and non-pregnant rats following RVFV-delNSs/NSm vaccination. (**A**) Experimental design for comparison of pregnant and non-pregnant humoral responses to RVFV-delNSs/NSm. Pregnant (E14, *n* = 6) and non-pregnant rats (*n* = 4) were vaccinated s.c. with 1 × 10^5^ PFU of RVFV-delNSs/NSm and serially bled at 0, 6, 14, 17, 21, 24, 28, and 31 days post-vaccination (d.p.v.). (**B**) Plasma anti-RVFV IgG titers measured by ELISA. Data are shown as the log_10_ geometric mean (GM) endpoint titer ± geometric standard deviation (GMSD). Dotted horizontal lines represent the limit of detection (1:100) and the upper limit of quantification (1:218,700). (**C**) Optical density values taken at 450 nm (OD_450_) of the 1:300 dilution. Data are shown as mean ± standard deviation. (**D**) Neutralizing antibody titers at 28 d.p.v. were determined by FRNT_80_. Data are shown as the log_10_ FRNT_80_. Bars represent the GM ± GMSD. Anti-RVFV IgG titers (**A**) between groups were compared using a mixed-effects analysis with Šidák’s multiple-comparisons test (*α* = 0.05, *P* = 0.10). Neutralizing antibody titers between groups (**D**) were compared using a two-tailed Mann-Whitney test (*P* = 0.91). Statistical tests yielding insignificant results are not shown. ND, none detected (below the LOD).

Immunological adaptations concurrent with pregnancy can impact the immune response to vaccination and should be considered when evaluating vaccine performance. To determine whether pregnancy impacts the timing, strength, and duration of immunogenicity induced by vaccination with RVFV-delNSs/NSm in rats, we compared antibody responses in age-matched, mid-gestation (embryonic day 14) pregnant (*n* = 6) and non-pregnant (*n* = 4) female rats. All rats were vaccinated with 1 × 10^5^ PFU of RVFV-delNSs/NSm, and longitudinal plasma samples were obtained to measure anti-RVFV IgG titers by ELISA (Fig. 1A). In pregnant rats, the placenta has a definitive structure by mid-gestation (E13–E15), and natural delivery occurs by approximately E23, corresponding to 9 days post-vaccination (d.p.v.). RVFV-delNSs/NSm vaccination induced a robust anti-RVFV IgG response in both groups by 12 d.p.v., which peaked between 14 and 17 d.p.v. (Fig. 1B). While anti-RVFV IgG titers reached detectable levels slightly sooner in pregnant rats (6 d.p.v.), the IgG response in this group appeared lower than in the non-pregnant animals, as measured by both geometric mean anti-RVFV IgG titer (Fig. 1B) and ELISA OD_450_ (Fig. 1C). More variability between pregnant animals was observed compared to their non-pregnant counterparts; however, there was no statistical difference in the magnitude of the anti-RVFV IgG response over time (mixed-effects analysis with Šidák’s multiple comparisons test; *α* = 0.05, *P* = 0.10).

Pregnancy is generally associated with Th2-type immunity (33, 34), so we measured RVFV-specific IgG1 and IgG2a titers, which serve as indicators of Th2- and Th1-mediated responses, respectively (35–37). When comparing endpoint titers, we found no differences in the induction of anti-RVFV IgG1 or IgG2a (two-tailed Mann-Whitney test; *P* = 0.91 and *P* = 0.076, respectively) between pregnant and non-pregnant rats following RVFV-delNSs/NSm vaccination (Fig. S1A and B). As a measurement of functional activity, we measured neutralization titers by FRNT_80_ at 28 d.p.v. (Fig. 1D). Both groups showed similar FRNT_80_ titers, with pregnant rats having more variability, but no significant differences were noted between the groups (two-tailed Mann-Whitney test; *P* = 0.91). Taken together, our data indicate that while subtle differences in the induction of IgG responses may exist between pregnant and non-pregnant states, neutralization capacity by 28 d.p.v. was comparable.

### High-dose, early gestation vaccination with RVFV-delNSs/NSm results in minimal dissemination of vaccine-derived virus and normal pregnancy outcomes

**Fig 2:**
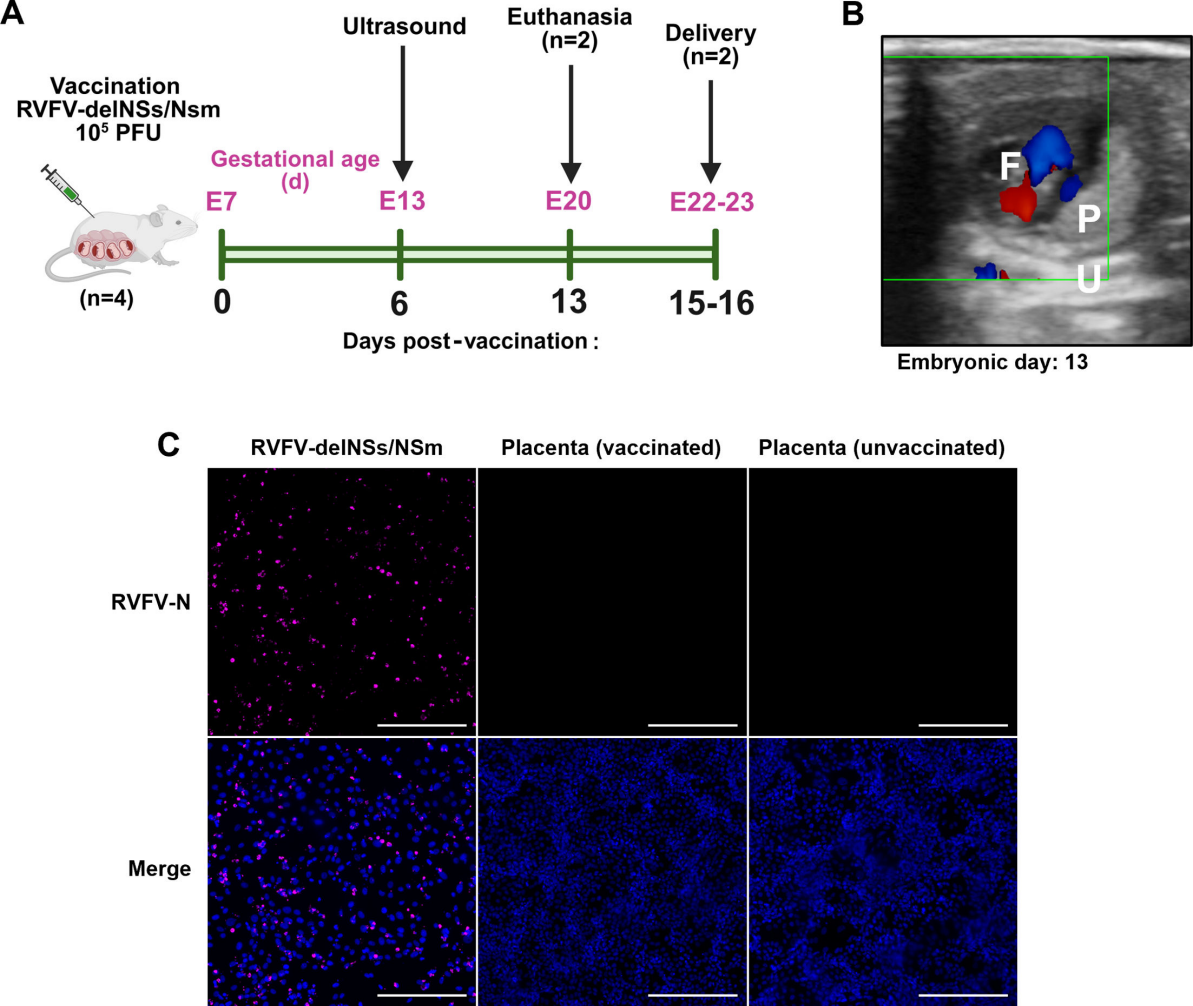
RVFV-delNSs/NSm vaccination is safe at a high dose during early gestation. (**A**) Experimental design for early gestation safety study. Pregnant rats (*n* = 4) were inoculated s.c. at E7 with 1 × 10^5^ PFU of RVFV-delNSs/NSm and euthanized either prior to (E20, *n* = 2) or following delivery (E22 and E23, *n* = 2) to collect maternal, placental, and fetal samples. Placentas were only available from dams euthanized prior to delivery. (**B**) Representative image of Doppler ultrasound used to verify pregnancy and assess fetal viability at E13 (6 d.p.v.). Red indicates blood flow toward the transducer, whereas blue indicates blood flow away from the transducer (F, fetus; P, placenta; and U, uterus). (**C**) Representative immunofluorescent images of Vero E6 cells inoculated with placental homogenate from RVFV-delNSs/NSm-vaccinated dams. Cells inoculated with RVFV-delNSs/NSm (MOI = 0.001) were used as a positive control, whereas cells inoculated with placental homogenate from unvaccinated dams were used as a negative control. Cells were fixed and stained for nuclei (DAPI; blue) and RVFV N (magenta) at 5 days post-inoculation. Images are at 10× magnification. Scale bar = 250 µm.

Our prior work with the Sprague-Dawley rat model focused on the outcomes of maternal RVFV vaccination or infection at mid-gestation (E14) (28, 30, 38). However, conflicting reports of RVFV LAV safety in pregnant livestock appear to depend on vaccine dose and gestational age, with higher rates of adverse outcomes observed in animals vaccinated during early gestation (16, 22, 39, 40). To address the safety profile of RVFV-delNSs/NSm when administered during early gestation at a high vaccine dose, pregnant rats (*n* = 4) were inoculated at E7 (prior to placenta maturation) with 1 × 10^5^ PFU of RVFV-delNSs/NSm (Fig. 2A). To examine pregnancy and fetal viability following maternal vaccination, we performed Doppler ultrasound at E13 (6 d.p.v.). Each pregnant rat had at least one living fetus, as indicated by blood flow to the conceptus (Fig. 2B). To assess the dissemination of RVFV-delNSs/NSm after vaccination, *n* = 2 dams were sacrificed at E20 (13 d.p.v.) to collect maternal and fetal samples. This time point ensured placentas could be harvested as dams typically consume them following birth. The remaining dams (*n* = 2) delivered between 15 and 16 d.p.v., and the general health of each pup was closely examined. All dams delivered normal litter sizes with no visibly apparent congenital abnormalities. We performed RT-qPCR to measure RVFV-delNSs/NSm-derived vRNA in the maternal and fetal samples collected at E20 (13 d.p.v.) and after delivery (15–16 d.p.v.). Low levels of vRNA just above the LOD were detected in a small number of samples collected after vaccination (Fig. S2). We attempted to isolate live virus by inoculating Vero E6 cells with placental homogenates that were nearing or above the LOD by RT-qPCR. Live virus could not be isolated from these tissues (Fig. 2C), suggesting that if minimal dissemination of RVFV-delNSs/NSm to the fetus occurred, there was limited clinical impact on the health of the offspring at delivery. RVFV-delNSs/NSm, therefore, appears to be safe for both dam and fetus when given at a high dose during early gestation.

### Vaccination protects against lethal maternal disease and *in utero* transmission of RVFV

**Fig 3:**
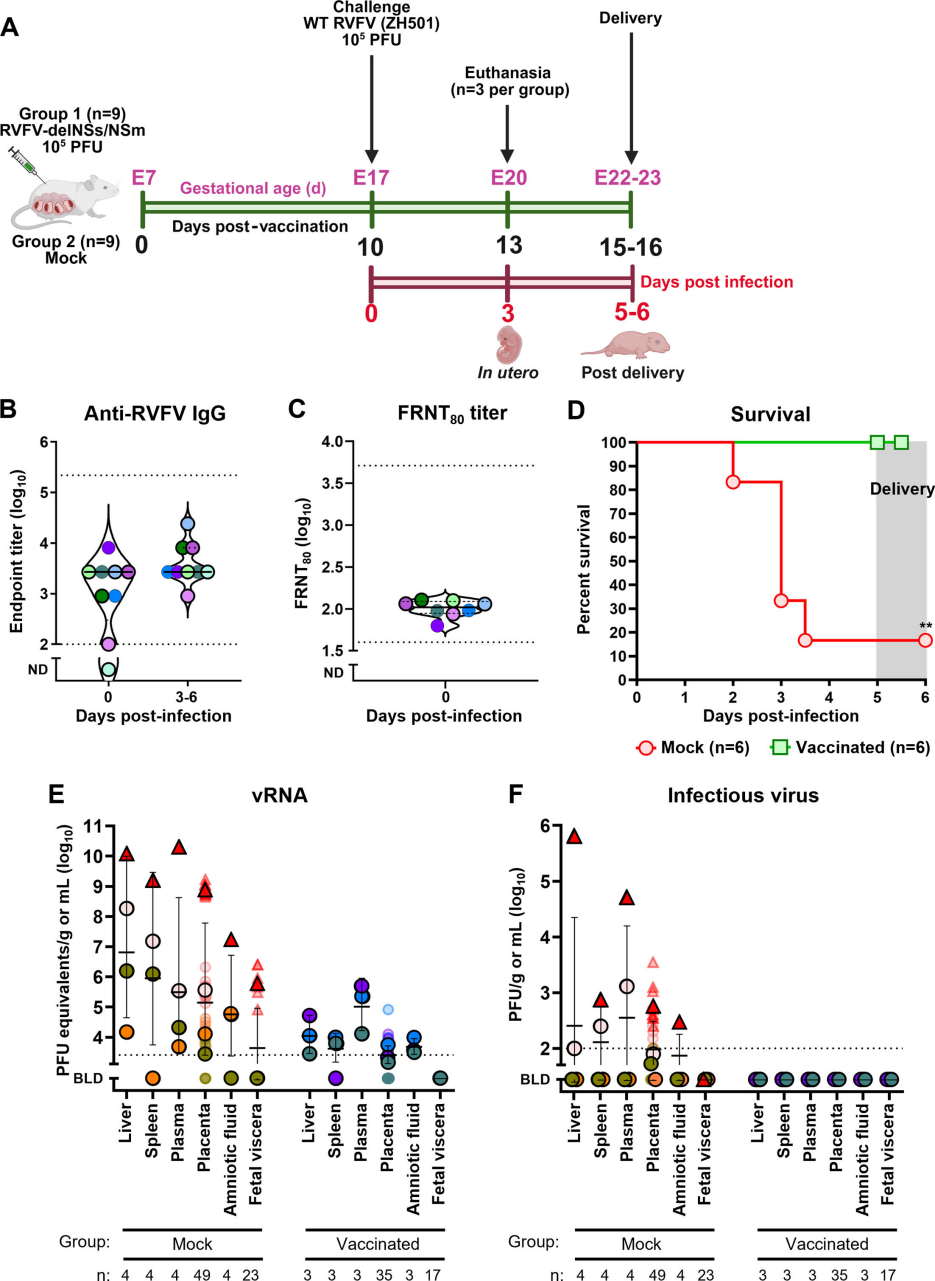
RVFV-delNSs/NSm-vaccinated dams and fetuses are protected from RVF and *in utero* transmission. (**A**) Experimental design for vaccination and challenge during pregnancy. Pregnant rats were vaccinated (s.c.) with 1 × 10^5^ PFU RVFV-delNSs/NSm (*n* = 9) or D2 media (mock, *n* = 9) at E7. At E17 (10 d.p.v.), all rats were bled and then challenged (s.c.) with 1 × 10^5^ PFU WT RVFV. At E20, *n* = 3 rats per group were euthanized to collect maternal, placental, and fetal samples. Surviving dams were monitored during delivery for stillbirths. Following delivery (between 5 and 6 d.p.i.), dams and pups were euthanized for sample collection. (**B**) Violin plot showing plasma anti-RVFV IgG titers in RVFV-delNSs/NSm-vaccinated dams prior to challenge (0 d.p.i., 10 d.p.v.) and at endpoint (3–6 d.p.i., 13–16 d.p.v.). Dotted lines represent the limit of detection (1:100) and the upper limit of quantification (1:218,700). (**C**) Violin plot showing neutralizing antibody titers prior to challenge (0 d.p.i., 10 d.p.v.) determined by FRNT_80_. Data are shown as the log_10_ FRNT_80_. One sample used for determining anti-RVFV IgG titers was unavailable for analysis by FRNT_80_. (**D**) Survival of RVFV-delNSs/NSm-vaccinated (*n* = 6) or mock-vaccinated dams (*n* = 6) following WT RVFV challenge. The shaded area indicates when surviving dams delivered their litters. Curve comparisons were performed using the Mantel-Cox test (**P = 0.0045). SuperPlots showing (**E**) vRNA and (**F**) infectious virus within maternal, placental, and fetal samples. Symbol colors correspond to individual dam:litter pairs. One animal in the mock-vaccinated group that succumbed to infection at 3 d.p.i. was included in this analysis (red colored triangles). Small symbols indicate the viral load in individual placental and fetal visceral samples from the same dam:litter pair. Larger symbols show the geometric mean (GM) viral load for all placental or visceral samples from that dam:litter pair. The dotted horizontal line represents the limit of detection of the q-RT-PCR assay (2,560 PFU equivalents/g or mL) or the VPA (100 PFU/g or mL). Error bars represent the GM (larger colored symbols) ± geometric standard deviation. BLD, below limit of detection; PFU, plaque-forming unit; vRNA, viral RNA.

A successful RVFV vaccine must overcome the challenge of preventing both maternal disease and *in utero* transmission should infection occur during pregnancy. Therefore, we sought to determine if vaccination during early gestation could protect against lethal challenge with WT RVFV (strain ZH501) during later gestation. On E7, a group of nine pregnant animals was vaccinated with RVFV-delNSs/NSm (1 × 10^5^ PFU) alongside nine pregnant mock-vaccinated controls (Fig. 3A). To confirm successful seroconversion, we collected plasma from each animal prior to challenge and measured anti-RVFV IgG titers by ELISA. By 10 d.p.v., eight out of nine vaccinated animals seroconverted with endpoint titers ranging from 1:100 to 1:8,100 (represented as log_10_ endpoint dilution) (Fig. 3B). Despite the wide range in anti-RVFV IgG titers, all animals tested had similar neutralization titers measured by FRNT_80_ (Fig. 3C). One sample used for determining anti-RVFV IgG titers was unavailable for analysis by FRNT_80_.

Using a stringent challenge dose (1 × 10^5^ PFU) of WT RVFV to assess vaccine efficacy, all rats regardless of seroconversion status were challenged at E17 (10 d.p.v.) (Fig. 3A) and monitored for signs of disease. By 3.5 days post-infection (d.p.i.), approximately 80% (5/6) of the mock-vaccinated dams developed severe disease and met euthanasia criteria; only one mock-vaccinated animal survived to delivery. In contrast, none of the RVFV-delNSs/NSm-vaccinated dams developed signs of disease regardless of seroconversion status prior to challenge (Fig. 3D). The single RVFV-delNSs/NSm-vaccinated dam that had no anti-RVFV IgG antibodies prior to challenge (10 d.p.v.) had fully seroconverted by 5 d.p.i. (15 d.p.v.) with an endpoint titer of 1:2,700 (represented as log_10_ endpoint dilution) (Fig. 3B). Plasma from this animal was not available for determination of FRNT_80_ titers.

We euthanized three animals per group at 3 d.p.i. (randomly selected) to assess the viral burden in maternal, placental, and fetal samples by RT-qPCR and/or viral plaque assay (VPA). These animals were excluded from the survival analysis (Fig. 3D). All available placentas and half of the fetuses from each litter (randomly selected) were collected for this analysis. One animal in the mock-vaccinated group that succumbed to infection at 3 d.p.i. was included in this analysis (indicated by red triangles). As expected, vRNA was detected in the maternal samples from mock-vaccinated dams. Infectious virus was detected by plaque assay in the maternal samples from two out of four mock-vaccinated dams euthanized at 3 d.p.i. (Fig. 3E and F).

In contrast to control animals, very low levels of vRNA were detected just above the limit of detection (LOD) in maternal and placental samples from dams vaccinated with RVFV-delNSs/NSm. However, no vRNA was detected in the viscera of matched fetuses (Fig. 3E). Infectious virus was not found in maternal, placental, or fetal samples from vaccinated animals (Fig. 3F). In conclusion, dams vaccinated with RVFV-delNSs/NSm during pregnancy survived stringent WT RVFV challenge and had reduced viral burden and *in utero* transmission.

### Maternal vaccination improves postnatal outcomes following WT RVFV challenge during pregnancy

**Fig 4:**
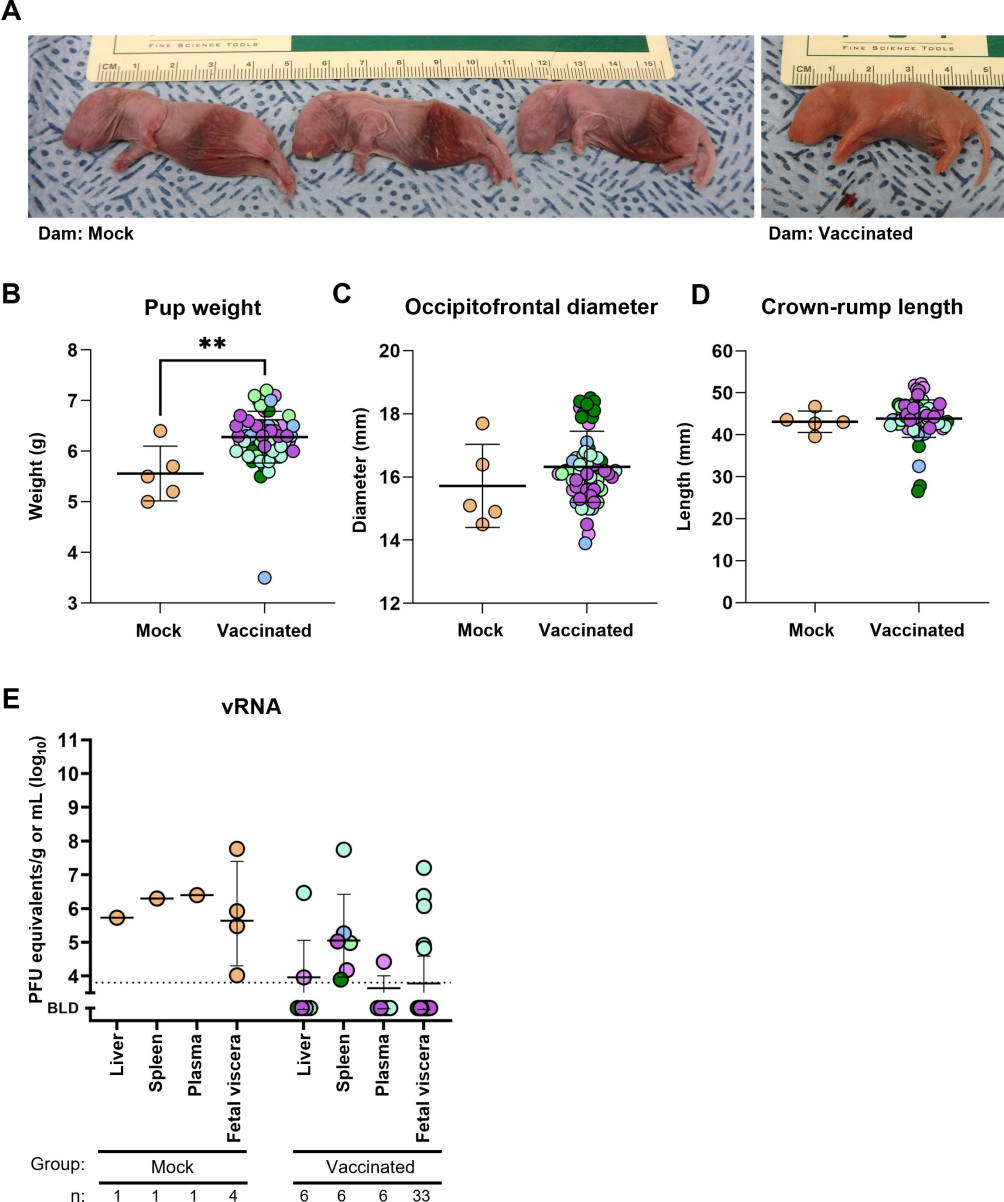
Impact of maternal RVFV-delNSs/NSm vaccination on offspring following WT RVFV challenge. (**A**) Representative images of pups born from mock (left, dead) or RVFV-delNSs/NSm-vaccinated dams (right, alive) following WT RVFV challenge on E17. Photos were taken immediately after delivery. (**B**) Weight, (**C**) occipitofrontal diameter, and (**D**) crown-rump length of pups born to surviving mock (*n* = 1 dam, 5 pups) or RVFV-delNSs/NSm-vaccinated dams (*n* = 6 dams; 66 pups). Measurements were only taken for live pups. Error bars represent the mean ± standard deviation. (**E**) vRNA within maternal and pup samples recovered upon delivery (5–6 d.p.i.). Each symbol color corresponds to individual dam:litter pairs. The dotted horizontal line represents the limit of detection of the q-RT-PCR assay (6,400 PFU equivalents/g or mL). Error bars represent the geometric mean ± geometric standard deviation. Pup measurements (**B–D**) were compared using unpaired, two-tailed Mann-Whitney tests (**P = 0.0047). Statistical tests yielding insignificant results are not shown.

A notable feature of RVFV during natural outbreaks in ruminants is the ability to cause severe congenital abnormalities, including musculoskeletal defects following maternal infection. In pregnant rats, RVFV-infected dams often deliver stillborn pups with gross physical anomalies even in the absence of maternal signs of disease (28). We closely examined the pups born to the surviving vaccinated or mock-vaccinated dams following RVFV challenge (Fig. 4A). Only 1/6 (17%) dams from the mock-vaccinated group survived to deliver offspring. From this litter, 5/8 pups (63%) were born alive and healthy with the remaining 3 pups (37%) found dead with apparent visceral hemorrhage (Fig. 4A; Fig. S3). In contrast, 66/70 pups (94%) from surviving vaccinated dams (*n* = 6) were born alive and healthy with only 4/70 pups (6%) found dead at delivery, with no apparent gross abnormalities. This is comparable to dams that were neither vaccinated nor infected and to dams that only received the vaccine (Fig. S3). In addition, pups from mock-vaccinated dams had lower birth weights (two-tailed Mann-Whitney test; *P* = 0.0047) and greater variability in occipitofrontal diameter, while crown-rump length remained similar compared to those from vaccinated dams (Fig. 4B through D).

We also measured viral loads in maternal (liver, spleen, and plasma) and offspring (viscera) samples from the surviving mock or vaccinated animals at delivery (5–6 d.p.i.). All maternal samples and half of the pups from each litter (randomly selected) were collected for this analysis. High levels of viral RNA were detected in all samples collected from the surviving mock-vaccinated dams and offspring. In contrast, we detected minimal levels of vRNA in samples collected from 5/6 vaccinated dam:offspring pairs. A single pup in the vaccinated group was not thriving and had an unusually low birth weight (3.5 g). However, there was no vRNA detected in the viscera. The dam that failed to seroconvert prior to challenge (10 d.p.v.) had levels of vRNA comparable to the mock-vaccinated dam (Fig. 4E). This dam delivered 11 living and 1 stillborn pup. None of these pups had apparent signs of gross congenital abnormalities. Additionally, we were unable to detect infectious virus in samples collected from either group. Altogether, our results show that a single, high dose of RVFV-delNSs/NSm given to rats during pregnancy is safe, immunogenic, and prevents lethal maternal disease, *in utero* transmission, and fetal demise after virulent WT RVFV challenge.

### Vaccination prior to pregnancy protects dams and fetuses from RVF during future pregnancy

**Fig 5:**
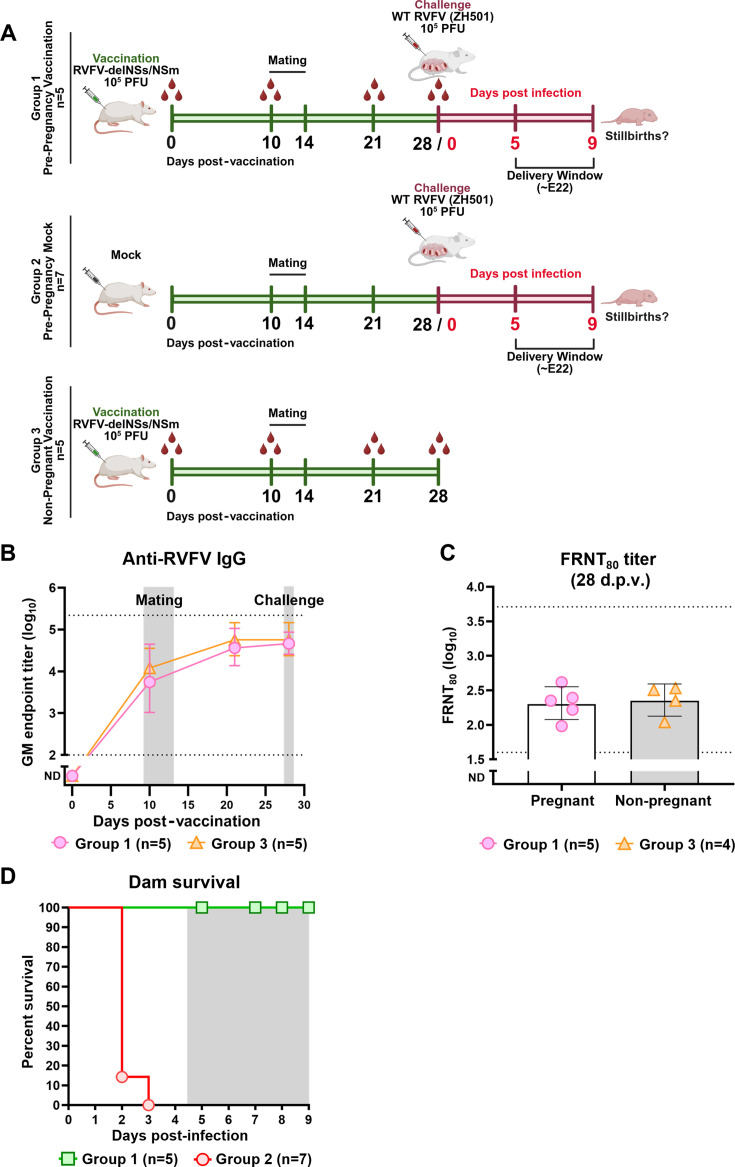
RVFV-delNSs/NSm vaccination prior to pregnancy protects against lethal RVF and vertical transmission. (**A**) Experimental design for assessing immunogenicity and efficacy of RVFV-delNSs/NSm vaccination prior to pregnancy. Female rats were vaccinated (s.c.) with 1 × 10^5^ PFU of RVFV-delNSs/NSm or D2 media (mock) and mated between 10 and 14 d.p.v. Pregnancy was confirmed after mating, and the female rats were divided into three groups. Group 1 consisted of pregnant dams vaccinated with RVFV-delNSs/NSm (*n* = 5). Group 2 consisted of mock-vaccinated pregnant dams (*n* = 7). Group 3 consisted of vaccinated females (*n* = 5) that failed to become pregnant after mating. All RVFV-delNSs/NSm-vaccinated rats (Groups 1 and 3) were serially bled at 0, 10, 21, and 28 d.p.v. Blood was not collected from mock-vaccinated dams (Group 2). At 28 d.p.v., pregnant RVFV-delNSs/NSm-vaccinated (Group 1; *n* = 5) and mock-vaccinated (Group 2; *n* = 7) dams were challenged (s.c.) with 1 × 10^5^ PFU WT RVFV (ZH501). For Groups 1 and 2, the experimental endpoint was when pregnant dams either met euthanasia criteria or delivered their offspring (5–9 d.p.i.). The experimental endpoint for Group 3 was 28 d.p.v. (**B**) Plasma anti-RVFV IgG titers from pregnant (Group 1; *n* = 5) or non-pregnant (Group 3; *n* = 5) female rats vaccinated with RVFV-delNSs/NSm prior to mating measured by ELISA. Blood was not collected from mock-vaccinated pregnant dams (Group 2). Data are shown as the log_10_ geometric mean (GM) endpoint titer ± geometric standard deviation (GMSD). Dotted horizontal lines represent the limit of detection (1:100) and the upper limit of quantification (1:218,700). (**C**) Neutralizing antibody titers in Group 1 and Group 3 dams prior to challenge (0 d.p.i., 28 d.p.v.) were determined by FRNT_80_. Data are shown as the log_10_ FRNT_80_. Bars represent the GM ± GMSD. One sample in Group 3 used for determining anti-RVFV IgG titers was unavailable for FRNT_80_. (**D**) Survival of pregnant RVFV-delNSs/NSm-vaccinated (Group 1; *n* = 5) and mock-vaccinated (Group 2; *n* = 7) dams. The shaded area shows the timing during which surviving dams delivered their litters.

Live-attenuated vaccines are generally contraindicated during pregnancy as they pose a theoretical risk to the mother and/or developing fetus. We sought to determine if vaccination prior to pregnancy could still protect against lethal maternal disease and *in utero* transmission if infection occurred during a subsequent pregnancy. Non-pregnant, female rats were vaccinated with 1 × 10^5^ PFU of RVFV-delNSs/NSm alongside mock-vaccinated controls. At 10 d.p.v., female rats were paired with male breeders for mating until 14 d.p.v. to increase the odds of successful pregnancy. Pregnancy was confirmed after mating, and the female rats were divided into three groups. Group 1 included pregnant dams vaccinated with RVFV-delNSs/NSm (*n* = 5), while Group 2 consisted of mock-vaccinated dams (*n* = 7). Vaccinated females that failed to become pregnant (*n* = 5) after mating were included in Group 3 (Fig. 5A).

To assess whether the onset of pregnancy impacted RVFV-delNSs/NSm immunogenicity, longitudinal plasma samples from RVFV-delNSs/NSm-vaccinated animals (Groups 1 and 3) were analyzed for anti-RVFV IgG titers by ELISA. Pregnant (Group 1) and non-pregnant (Group 3) females had nearly identical anti-RVFV IgG responses across all time points (Fig. 5B). Similarly, neutralization titers prior to challenge (28 d.p.v.) were comparable between Groups 1 and 3 as measured by FRNT_80_ (Fig. 5C), indicating that the onset of pregnancy did not impact RVFV-delNSs/NSm immunogenicity. One sample from Group 3 used for determining anti-RVFV IgG titers at 28 d.p.v. was unavailable for analysis by FRNT_80_.

To determine the efficacy of RVFV-delNSs/NSm vaccination prior to pregnancy, vaccinated (Group 1, *n* = 5) and mock-vaccinated (Group 2; *n* = 7) pregnant dams were challenged with 1 × 10^5^ PFU of WT RVFV at 28 d.p.v. (Fig. 5A). Vaccinated females that failed to become pregnant (Group 3) were excluded from the survival analysis. Given that the mating period lasted for three full days, the gestational ages of the dams on the day of challenge ranged from E14 to E17. All RVFV-delNSs/NSm-vaccinated dams (Group 1; *n* = 5) survived to deliver their pups between 5 and 9 d.p.i. In contrast, all mock-vaccinated dams (Group 2; *n* = 7) succumbed to RVF by 3 d.p.i. (Fig. 5D). Consistent with our findings when vaccination occurred during pregnancy, only 2/58 pups (3%) from RVFV-delNSs/NSm-vaccinated dams were found dead at delivery, and we observed no gross congenital abnormalities in any of these pups (Fig. S3). Furthermore, no vRNA was detected in maternal or pup samples collected at necropsy which occurred immediately after delivery (5–9 d.p.i.) (Fig. S4). In conclusion, our findings indicate that RVFV-delNSs/NSm vaccination prior to pregnancy protects against lethal maternal disease and *in utero* transmission if infection occurs during a future pregnancy.

## DISCUSSION

RVFV sporadically causes devastating animal and human disease in Africa and has significant potential to expand into non-endemic regions. The abortogenic potential of RVFV in pregnant livestock has been well documented since the virus was discovered in 1931 (41). However, the recognition of this phenomenon in humans has emerged only recently and remains understudied. Two early, retrospective serosurveys found little evidence of an association between RVFV infection and fetal loss in pregnant individuals (42, 43). These studies were severely limited by the lack of serology for anti-RVFV IgM to confirm active or recent infections as well as assuming the study participants had no prior RVFV exposure. Regardless, the authors of both studies emphasized caution and the need for more epidemiological data given the proclivity of RVFV to cause fetal death in several animal species. Indeed, two decades later, case reports during RVFV outbreaks in the 2000s provided evidence of *in utero* transmission of RVFV in humans (11, 12). Despite these findings, there remains a lack of comprehensive epidemiological data regarding *in utero* transmission of RVFV in humans. In 2016, a cross-sectional study with 130 pregnant Sudanese individuals found that infection with RVFV during pregnancy correlated with a fourfold increase in late-gestation miscarriage rates (13). Further, laboratory experiments have shown the ability of RVFV to infect the human placenta (28, 44). These data indicate that *in utero* transmission of RVFV in humans is likely an underestimated manifestation of disease that could have severe consequences for both the mother and the developing fetus.

The emergence of Zika virus (ZIKV) in the Americas highlighted how the introduction of viruses to new regions can have dramatic effects in vulnerable populations such as pregnant individuals. Prior to the 2015 epidemic in Latin America, evidence linking ZIKV to congenital disease was limited (45). However, by 2016, the association between congenital ZIKV and microcephaly was firmly established, prompting an intense global response (46). More recently, the spread of novel Oropouche virus (OROV) reassortants in Brazil has been associated with fetal infection, fetal demise, and congenital defects despite having been previously undocumented (47–49). Laboratory experiments support these clinical observations, demonstrating the permissivity of the maternal-fetal interface to OROV infection in human tissue explants (50) and in mice (51, 52). Given that OROV and RVFV are phylogenetically related, *in utero* transmission may represent a shared characteristic of bunyaviruses. Notably, RVFV appears to replicate more efficiently in human placental tissues compared to OROV (28, 44), suggesting that RVFV poses an equal or greater risk to pregnant individuals. Therefore, developing safe and effective RVFV vaccines for emerging outbreaks to protect high-risk populations such as pregnant individuals is critical.

Considerable progress has been made in developing RVFV vaccines. Unfortunately, none are licensed for human use. The live-attenuated Smithburn and Clone-13 vaccines are still widely used in livestock to control the spread of RVFV in endemic regions (14). A major drawback of these two vaccines is they retain partial virulence in pregnant and young animals (15, 16), thus preventing their widespread use including in humans. Initial studies with another RVFV LAV, MP-12, demonstrated safety in ewes during late gestation (70–110 days of pregnancy) with doses ranging from 5 × 10^3^ to 5 × 10^5^ PFU (17, 18). However, a follow-up study in early gestation ewes (28–56 days of pregnancy) with a higher dose of vaccine (1 × 10^6^ PFU) resulted in stillborn lambs with congenital deformities (22). These findings raise concerns regarding the suitability of MP-12 as a human vaccine. Promising next-generation LAV candidates based on the MP-12 backbone, including RVax-1 and arMP-12ΔNSm21/384, are in development (53–56). The arMP-12ΔNSm21/384 vaccine showed poor safety in early gestation ewes (40), and RVax-1 was recently tested in pregnant rodents, where vaccination was safe in pregnant rats but not in pregnant mice (57). Therefore, future work addressing the safety and efficacy of these vaccines during pregnancy would support their advancement as human RVFV vaccine candidates. Other human LAV candidates like hRVFV-4s are in clinical trials and have shown safety and efficacy in pregnant animal models (58, 59), indicating their potential for use in pregnant individuals.

Vaccine platforms that do not contain live virus are generally considered to have a more acceptable safety profile during pregnancy than live-attenuated vaccines. Alternative RVFV vaccine platforms that have undergone recent clinical trials are the chimpanzee adenovirus vectored vaccine, ChAdOx1 RVF, and the formalin inactivated vaccine, TSI GSD 200. The lack of efficacy in pregnant animals and requirement of multiple doses to achieve desirable immunogenicity in humans (60, 61) makes the TSI GSD 200 vaccine a poor candidate to protect high-risk populations like pregnant individuals. On the other hand, a single dose (1 × 10^9^ infectious units) of ChAdOx1 RVF was safe in pregnant ewes and does and completely protected ewes from virulent RVFV challenge (62). Some maternal and fetal samples from vaccinated animals contained low levels of viral RNA and live virus was isolated from a single placentome following virulent challenge, indicating incomplete protection in pregnant goats (62). While ChAdOx1 RVF is a promising alternative to live-attenuated vaccines in pregnant populations, rare occurrences of vaccine-induced thrombotic thrombocytopenia (VITT) have been reported in individuals who received adenovirus-vectored COVID-19 vaccines (63–65). Whether this poses an increased risk for individuals with hypercoagulable states, such as pregnancy, remains debated (66, 67).

The live-attenuated RVFV-delNSs/NSm vaccine used in this study has proven safe and efficacious in several animal models, including early gestation pregnant sheep (23–25). Given conflicting safety reports for other RVFV LAVs during early gestation, our study extends these findings by evaluating the safety of a high dose (1 × 10^5^ PFU) of RVFV-delNSs/NSm in early gestation (E7) pregnant rats. A limitation of these analyses is the small number of animals used due to logistical considerations when working at ABSL-3. Very low levels of vRNA were detected in a small number of samples collected after vaccination, although we found no evidence of infectious virus nor congenital abnormalities resulting from vaccination itself, underscoring a favorable safety profile in pregnant animals. Future work will be directed toward more subtle indicators of maternal vaccine safety including the impact on fertility and the long-term developmental health of the offspring.

Furthermore, we showed that pregnant rats vaccinated with RVFV-delNSs/NSm were protected from lethal RVF, *in utero* transmission, and fetal demise following WT RVFV challenge. Due to the short gestational length of rats, WT RVFV challenge occurred at 10 d.p.v., allowing minimal time for dams to fully develop an adaptive response to vaccination. This likely explains the high variability in anti-RVFV IgG titers and why one dam had not seroconverted by the day of challenge. Regardless, at this time point, all dams for which samples were available had detectable neutralization titers. Following challenge, this dam’s anti-RVFV IgG titer increased rapidly, reaching detectable levels by 5 d.p.i. These findings suggest that RVFV-delNSs/NSm vaccination can potentially confer protection even without a detectable anti-RVFV IgG response. This is supported by evidence that shows low levels of neutralizing antibodies are protective against WT RVFV (30, 68). Alternatively, robust induction of innate immune mediators is associated with survival from RVFV infection and may have contributed to the protection observed in this dam (69–71).

When assessing post-birth outcomes, some analyses were limited by the lethality of WT RVFV in unvaccinated pregnant rats, leaving only one surviving mock-vaccinated dam:litter pair for comparison of post-birth outcomes. Despite this limitation, litters from vaccinated dams had rates of stillbirth and congenital abnormalities that were no different from unvaccinated control pregnant animals.

The distinct immunological milieu of pregnancy warrants consideration of the potential impact pregnancy has on vaccine immunogenicity and efficacy (72). Many studies indicate that pregnant individuals mount humoral responses as robust as their non-pregnant counterparts (73–75). While comparing humoral responses to RVFV-delNSs/NSm vaccination in pregnant and non-pregnant rats, we observed similar kinetics and strength of antibody responses; however, anti-RVFV IgG titers were more variable when vaccination occurred during pregnancy. A limitation of this analysis is the use of a single vaccine dose at one point in gestation. Pregnant rats are widely used to study placentation and for pre-clinical toxicological assessment during vaccine and therapeutic development. However, there are relevant differences in pregnancy physiology between rats and humans, including placental architecture, transfer of maternal immunity, and gestational length (76). Therefore, our findings may not fully reflect the kinetics of anti-RVFV antibody responses in pregnant people. Testing lower doses across various points in gestation may identify subtle differences in vaccine responses during pregnancy in the rat model. While outside the scope of this study, some evidence indicates subtle differences in the functionality of maternal antibodies may exist, including Fc binding and effector function, and cross-reactivity to heterologous antigens (77, 78). Cellular immunity is also altered during normal pregnancy, favoring a less inflammatory, Th2 phenotype, which may impact vaccine responses (33). Therefore, additional studies investigating potential differences in vaccine responses between pregnant and non-pregnant individuals are urgently needed to expand access to RVFV vaccines for this vulnerable population.

Finally, the exclusion of pregnant individuals from vaccine clinical trials and subsequent lack of safety and immunogenicity data remains a considerable obstacle limiting access to vaccines (79). During the COVID-19 pandemic, 25% of low- and middle-income countries advised against COVID-19 vaccination during pregnancy, often citing a lack of clinical trial data (80). A concern with the use of LAVs during pregnancy is the theoretical risk posed to the fetus. Therefore, LAVs are generally contraindicated during pregnancy; however, they may be recommended when the benefits of vaccination outweigh the risks, as is the case for Yellow Fever (81). One way to circumvent the use of LAVs in this population is to vaccinate individuals of child-bearing age prior to pregnancy. While rodents do not fully recapitulate human pregnancy, our data indicate RVFV-delNSs/NSm vaccination prior to pregnancy potentially remains effective throughout gestation, providing an alternative approach to protect pregnant individuals and their fetuses from RVFV. Given the exceptional risk RVFV poses to pregnant individuals and the lack of licensed vaccines, the inclusion of pregnant individuals in RVFV vaccine clinical trials could potentially protect this population during future emergency outbreak responses.

This study provides evidence that vaccination of rats with RVFV-delNSs/NSm prior to and during pregnancy is immunogenic, has a high safety profile, and provides complete protection against lethal RVF. RVFV likely poses an underestimated risk to pregnant individuals, and vaccination prior to or during pregnancy has the potential to reduce human disease burden in this vulnerable population. As several RVFV vaccines have reached clinical trials, the inclusion of pregnant individuals should be considered to provide adequate safety and immunogenicity data ahead of emergent outbreaks.

## MATERIALS AND METHODS

### Viruses

Stuart Nichol (CDC, Atlanta, GA, USA) provided the original reverse genetics-derived WT RVFV (ZH501) used in this study (82). RVFV-delNSs/NSm used for vaccination and the RVFV-delNSs/NSm encoding a ZSG tag in place of the RVFV-NSs gene (RVFV-delNSs/NSm-ZSG) used for neutralization assays were generated by reverse genetics as previously described (83). This system was kindly provided by Cesar Albarino (CDC). Virus was propagated using Vero E6 cells (ATCC, CRL-1586) cultured in D2 media (Dulbecco’s modified Eagle’s medium [DMEM] containing 2% FBS, 1% penicillin-streptomycin, and 1% l-glutamine), following standard cell culture procedures. The viral stock titer was determined by viral plaque assay (VPA) as previously described (28).

### Animal experiments

Non-pregnant and time-mated female Sprague-Dawley rats (6–10 weeks old) were obtained from Envigo Laboratories (Inotiv). Rats were delivered to the University of Pittsburgh, single-housed in temperature-controlled rooms with a 12 h day/12 h night light schedule. Food and water were provided *ad libitum*. Data compiled from several independent experiments are presented in this manuscript. The sample sizes chosen for animal experiments are based on previously published work (28, 29, 38).

For all ultrasounds and procedures, rats were anesthetized with vaporized isoflurane (IsoThesia, Henry Schein) and placed on a heating pad (E-Z Systems, HB-163XL) at 37°C to maintain body temperature. Mock vaccination (D2 media), RVFV-delNSs/NSm vaccination, and challenge with WT RVFV (diluted to desired PFU in D2 media) were performed via subcutaneous injection (200 µL total). Longitudinal blood sampling was performed via lateral saphenous vein puncture (opposite side of vaccination), and Nair was used to remove fur from the hind limbs when necessary. Where applicable, blood was also collected from rats at euthanasia by cardiac puncture. All blood was collected in EDTA-treated tubes and centrifuged for plasma collection. Across experiments, time points may have varying sample sizes due to limited sample quantity or asynchronous time-point collections. Across all experiments, maternal samples included liver, spleen, and plasma. Fetal/pup samples included viscera. Placentas are comprised of both maternal- and fetal-derived tissue. All samples were taken immediately upon necropsy and stored at −80°C prior to downstream virological analyses.

For WT RVFV challenge studies, dams were implanted with programmable temperature transponders (Avidity Science, TP-1000) subcutaneously between the shoulders and monitored twice daily for clinical signs of disease, changes in body temperature, and weight loss. Animals that met euthanasia criteria or were selected for planned euthanasia were humanely euthanized according to established IACUC guidelines. Animals selected for planned euthanasia were chosen randomly and excluded from survival analyses. Surviving dams delivered their pups between E22 and E23, and the proportion of pups born dead or alive was recorded. Pups were closely examined for signs of gross congenital abnormalities. For evaluation of post-birth outcomes, weight, occipitofrontal diameter (snout to the most prominent portion of the occipital bone), and crown-rump length (top of the head to base of tail) measurements were taken for each pup.

For pre-pregnancy vaccination studies, female rats were paired 1:1 with male breeders for three full days (between 10 and 14 d.p.v.). On day 14 post-vaccination, females were separated and single-housed. Pregnancy was initially confirmed by weight gain after mating and once again upon necropsy of the animal. The female rats were divided into three groups based on pregnancy or vaccination status. Group 1 included pregnant dams vaccinated with RVFV-delNSs/NSm, while Group 2 consisted of mock-vaccinated dams. Some vaccinated females failed to become pregnant after mating and were included in Group 3 (Fig. 5).

As the focus of this study was vaccine immunogenicity, safety, and efficacy in pregnant rats, only data from female rats were used. Therefore, sex differences were not analyzed as part of this study; however, data from both male and female pups were included throughout.

### Ultrasound

When dams reached E13 (6 d.p.v.), pregnancy and fetal viability were evaluated using a portable, color Doppler ultrasound machine (Eden Acclarix, AX3). Briefly, animals were anesthetized, and a small volume of ultrasound gel was applied to the transducer and the abdomen of the dam. The transducer was gently placed on the abdomen of the dam and traced along the uterine horns to obtain colorized sonographs using the Doppler feature.

### ELISA

Plasma collected in this study was used for an indirect RVFV whole-lysate ELISA (26, 30, 84). For semi-quantification of total anti-RVFV IgG, anti-RVFV IgG1, and anti-RVFV IgG2a, 96-well MaxiSorp plates (ThermoFisher, 442404) were coated with 100 µL of whole-cell lysate from RVFV-delNSs/NSm infected Vero E6 cells and incubated overnight at 4°C. Whole-cell lysate from uninfected Vero E6 cells was used as a negative control. Lysates were used at 1:1,000 diluted in sterile PBS. The following day, plates were blocked with 5% non-fat, dry milk in PBST (PBS, 0.1% Tween-20) for 1 h at 37°C. Plasma was serially diluted in block and added to the plates in duplicate. Plasma from each rat collected prior to vaccination (0 d.p.v.) was used as a negative control. Plates were washed three times with PBST and incubated at 37°C for 1 h with either HRP-labeled goat anti-rat IgG (Invitrogen, 31470), IgG1 (Invitrogen, PA1-84708), or IgG2a (Invitrogen, PA1-84709) at 1:5,000 diluted in block. Plates were washed again three times with PBST and incubated for 10 min at room temperature with TMB substrate (SeraCare, 5120-0047). Reactions were stopped using TMB Stop Solution after 10 min development time (SeraCare, 5150-0021), and plates read at 450 nM. The plasma dilutions tested ranged from 1:100 to 1:218,776 and constituted the LOD and upper limit of quantification (ULOQ), respectively (represented as log_10_). Endpoint titers were defined as the highest plasma dilution with an optical density (OD) exceeding the average of the negative control wells plus two standard deviations after background subtraction of the uninfected Vero E6 cells. If all negative control wells resulted in negative values after background subtraction, three times the standard deviation of the negative control wells was used to define the endpoint titer. True negative values (defined as none detected; ND) were assigned a nominal value corresponding to ½ the LOD.

### Focus reduction neutralization assay

Plasma collected in this study was used for a focus reduction neutralization test (26, 30, 85). Plasma was heat-inactivated and serially diluted in duplicate and incubated at 37°C for 1 h with 100–200 focus-forming units of RVFV-delNSs/NSm-ZSG. Virus only or plasma from unvaccinated rats were used as negative controls. The plasma dilutions tested ranged from 1:40 to 1:5,120 and constituted the LOD and ULOQ respectively (represented as log_10_). After incubation, 100 µL of the plasma/virus mixture was added to 96-well tissue culture plates containing 2 × 10^4^ Vero E6 cells/well (ATCC, CRL-1586) and incubated at 37°C for 1 h. After incubation, the inoculum was removed and 100 µL of 1.5% carboxymethylcellulose (CMC) (Sigma C4888-500G) was added to each well and incubated at 37°C for approximately 18 h. CMC was then removed, and wells were washed once with sterile PBS. Plates were then fixed with 37% formaldehyde and imaged using a Cytation 5 cell imaging multimode reader (Agilent, BioTek). Foci were counted using Gen5 (Agilent, BioTek) or ImageJ software, and the plasma dilution at which 80% virus neutralization occurred was interpolated by non-linear regression and reported as the log_10_ FRNT_80_.

### RNA isolation and RT-qPCR

Tissue samples were placed in D2 media (wt/vol) and homogenized. Tissue homogenate or plasma was inactivated by mixing 1:10 in TRIzol reagent (Invitrogen) prior to removal from the RBL. RNA isolation and one-step RT-qPCR targeting the L segment were performed as previously described (28). Data were reported as PFU equivalents/g or mL (log_10_) based on a standard curve titrated from an RVFV stock of known titer. The LOD corresponds to the lowest standard curve dilution. Both true negative and detectable values below the LOD (defined as below limit of detection; BLD) were assigned a nominal value corresponding to ½ the LOD.

### Viral plaque assay

Tissues homogenates or liquid samples (i.e., plasma and amniotic fluid) were serially diluted in duplicate and titrated on monolayers of Vero E6 cells (ATCC, CRL-1586). Inoculated cells were incubated for 1 h at 37°C, and 3 mL of agarose overlay (minimum essential medium, 2% FBS, 1% penicillin/streptomycin, 1.5% HEPES buffer, and 0.8% SeaKem agarose) was added to each well. Cells were fixed with 37% formaldehyde after a 3-day incubation at 37°C and stained with 0.1% crystal violet. Plaques were manually enumerated and reported as PFU/g or mL (log_10_). The LOD is based on the lowest number of countable plaques. Both true negative and detectable values below the LOD (defined as below limit of detection; BLD) were assigned a nominal value corresponding to ½ the LOD.

### Virus isolation assay

Placental homogenates that had vRNA levels nearing or above the LOD by RT-qPCR were selected for virus isolation. Homogenates were clarified and diluted 1:1 in D2 media after which, 100 µL was used to inoculate duplicate monolayers of Vero E6 cells grown in 12-well plates on glass coverslips (2 × 10^5^ cells/well). RVFV-delNSs/NSm diluted in D2 to an MOI of 0.001 was used as a positive control. After a 1-h adsorption period at 37°C, 1 mL of D2 media was added to each well and incubated for 5 days. Supernatant was collected from each sample at 0, 3, and 5 d.p.i. for RT-qPCR analysis as described above. After 5 days, the inoculum was removed, and cells were fixed with 4% paraformaldehyde for immunofluorescence staining. For immunofluorescence assays, cells were permeabilized with 0.1% Triton X-100 diluted in PBS, washed three times with 2.5% normal goat serum, and blocked with 5% normal goat serum for 1 h at room temperature. Cells were then washed three times and stained for 2 h at room temperature with rabbit anti-RVFV N IgG (Genescript: 05540) diluted to 1:500 in block. Cells were then washed three times and stained for 1 h at room temperature with Alexa Fluor 594-conjugated goat anti-rabbit IgG (Invitrogen, A211012) diluted to 1:1,000 in block. Cells were then washed three times and incubated with Hoechst/DAPI (1:1,000 in PBS) for 45 s. Cells were then washed twice with PBS and coverslips were adhered to glass slides at 4°C overnight using gelvatol. The next day, slides were imaged using a Leica microscope at 10× magnification. Images were subsequently processed using ImageJ and PowerPoint.

### Statistics

All data visualization and associated statistical analyses included in this manuscript were performed using GraphPad Prism. The statistical test applied to each figure is included in the text and in the corresponding figure legends.

## Data Availability

All data are available within the main text or supplementary materials files.

## SUPPLEMENTAL MATERIAL

Supplemental figures mbio.00253-26-s0001.pdf Fig. S1-S4.

ASM does not own the copyrights to Supplemental Material that may be linked to, or accessed through, an article. The authors have granted ASM a non-exclusive, world-wide license to publish the Supplemental Material files. Please contact the corresponding author directly for reuse.
