# Supplementary material for: Maternal vaccination protects dams and prevents in utero transmission of Rift Valley fever virus in rats
Source: mBio. 2026 Jun 22;17(8):e00253-26. doi: 10.1128/mbio.00253-26 (PMC13464346; doi:10.1128/mbio.00253-26)
Supplement: Supplementary file 1 [file mbio.00253-26-s0001.pdf]

Hertel et al., Supplementary Figure 1

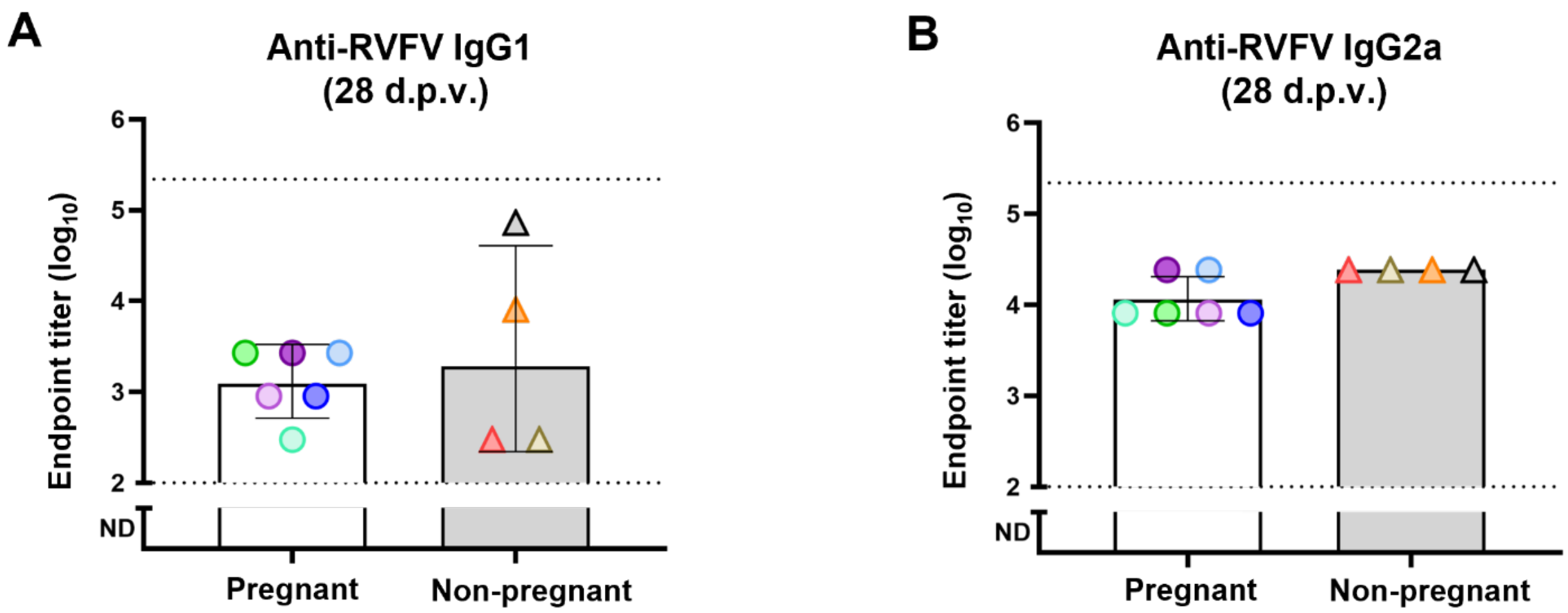

Fig S1. IgG subclasses induced by RVFV-delNSs/NSm vaccination. (A) Plasma anti-RVFV IgG1 and **(B)** anti-RVFV IgG2a titers in pregnant (n=6) and non-pregnant rats (n=4) at 28 d.p.v. were measured by ELISA. Data are shown as the $\log_{10}$ endpoint titer. Dotted horizontal lines represent the limit of detection (1:100) and upper limit of quantification (1:218700). Bars represent the geometric mean ± the geometric standard deviation. Anti-RVFV IgG1 **(A)** and IgG2a **(B)** titers between groups were compared using two-tailed, Mann-Whitney tests (p=0.91 and p=0.076 respectively). Statistical tests yielding insignificant results are not shown. ND, not detected (below the LOD).

Hertel et al., Supplementary Figure 2

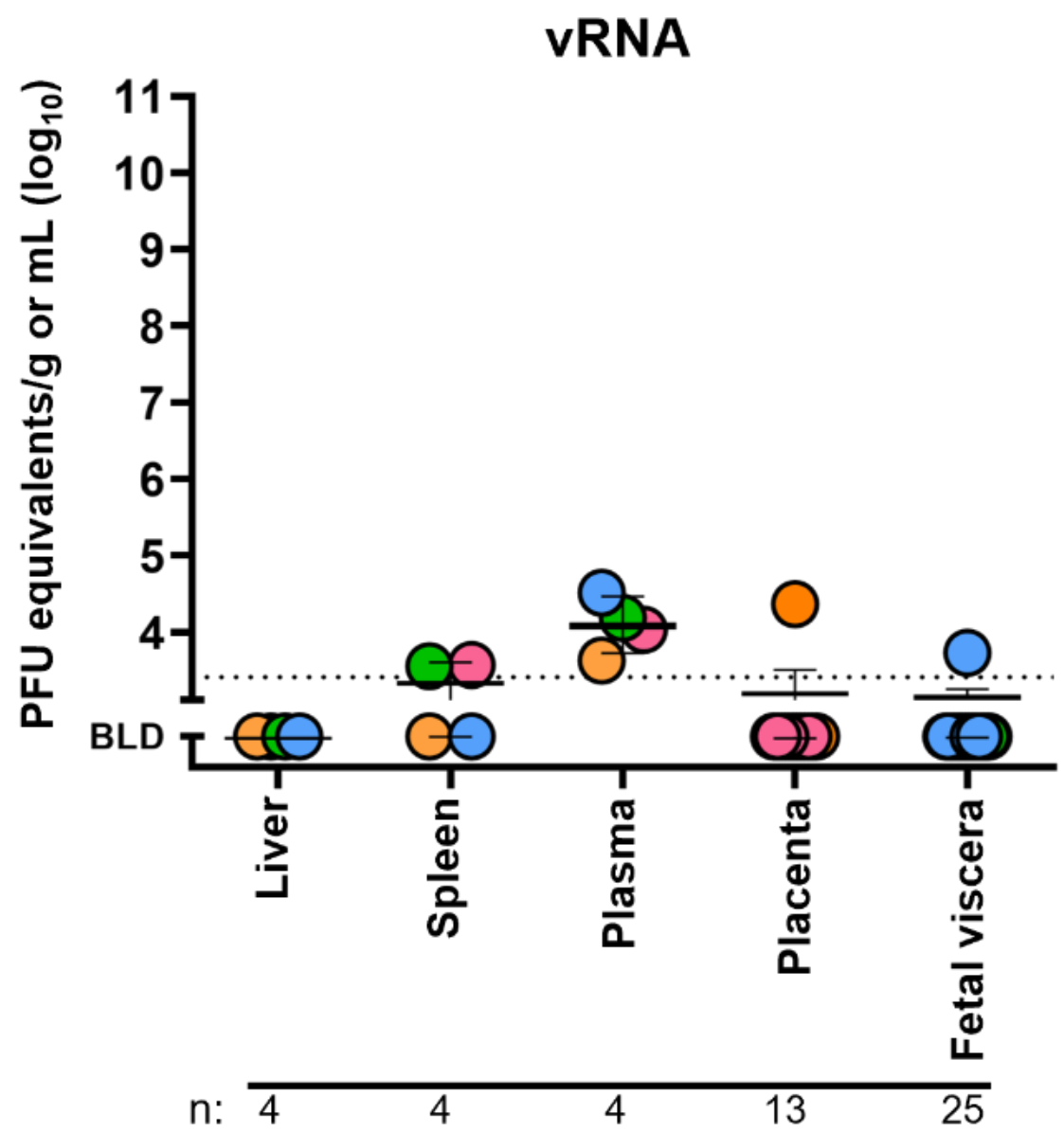

Fig S2. Minimal vRNA detected in maternal/fetal tissues following early gestation vaccination with RVFV-delNSs/NSm. vRNA within maternal, placental, and fetal samples. Each symbol color corresponds to individual dam:litter pairs. The dotted horizontal line represents the limit of detection of the q-RT-PCR assay (2560 PFU equivalents/g or mL). Error bars represent the geometric mean ± the geometric standard deviation. vRNA, viral RNA; PFU, plaque-forming unit; BLD, below limit of detection.

Hertel et al., Supplementary Figure 3

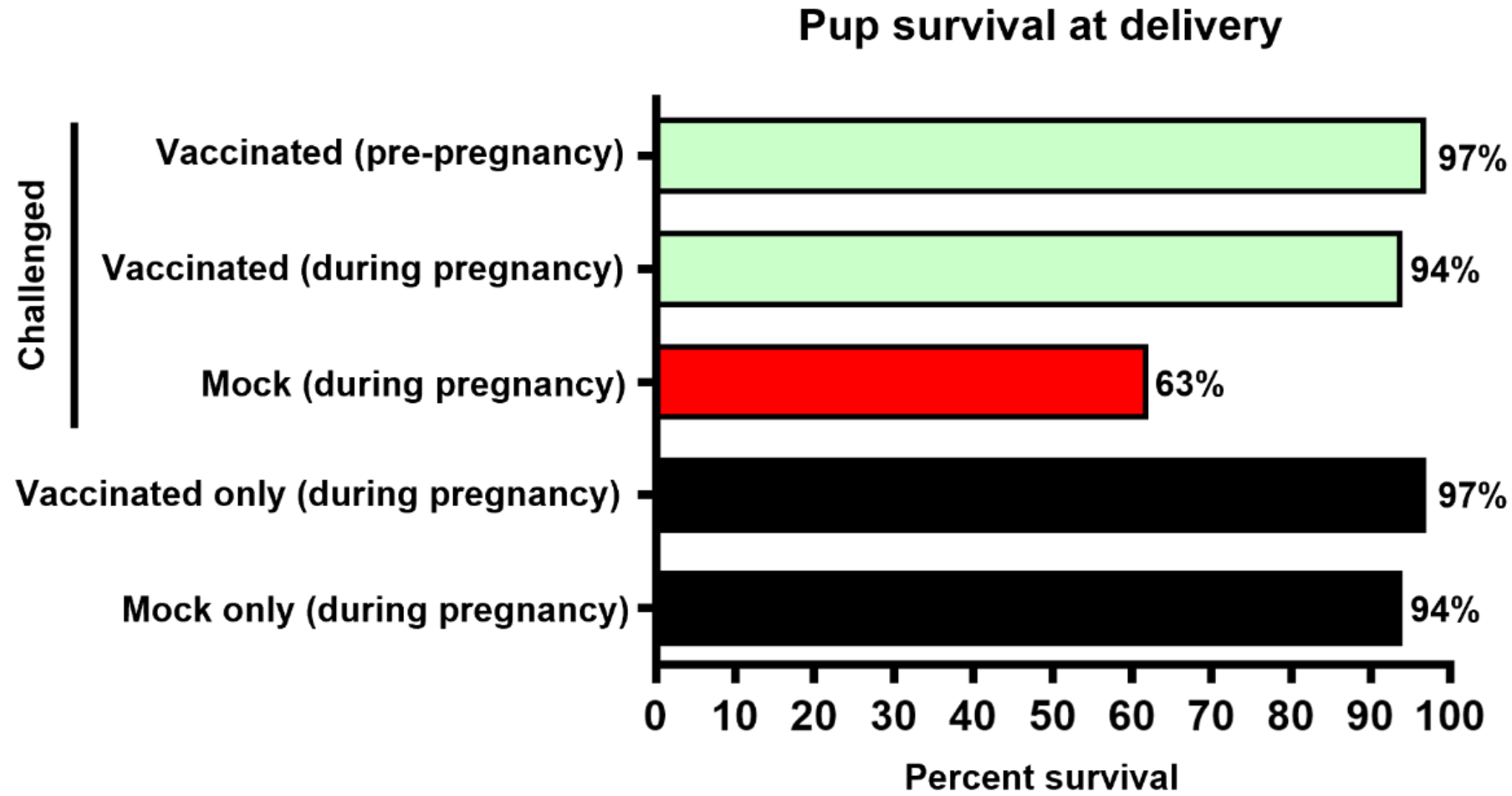

Fig S3. Survival of pups born to RVFV-delNSs/NSm vaccinated or mock vaccinated dams. Percent survival of pups at delivery across all experiments. “Mock only (during pregnancy)” represents pup survival from dams that only received a mock vaccination during pregnancy (n=8 dams, 88 pups), compiled from 3 independent experiments. “Vaccinated only (during pregnancy)” represents pup survival from dams vaccinated solely with RVFV-delNSs/Nsm during pregnancy (n=2 dams, 30 pups). The remaining groups show survival of pups born to dams that were either mock vaccinated (n=1 dam, 8 pups) or vaccinated with RVFV-delNSs/NSm (n=6 dams, 70 pups) during pregnancy, or vaccinated prior to pregnancy (n=5 dams, 58 pups) and subsequently challenged with WT RVFV.

Hertel et al., Supplementary Figure 4

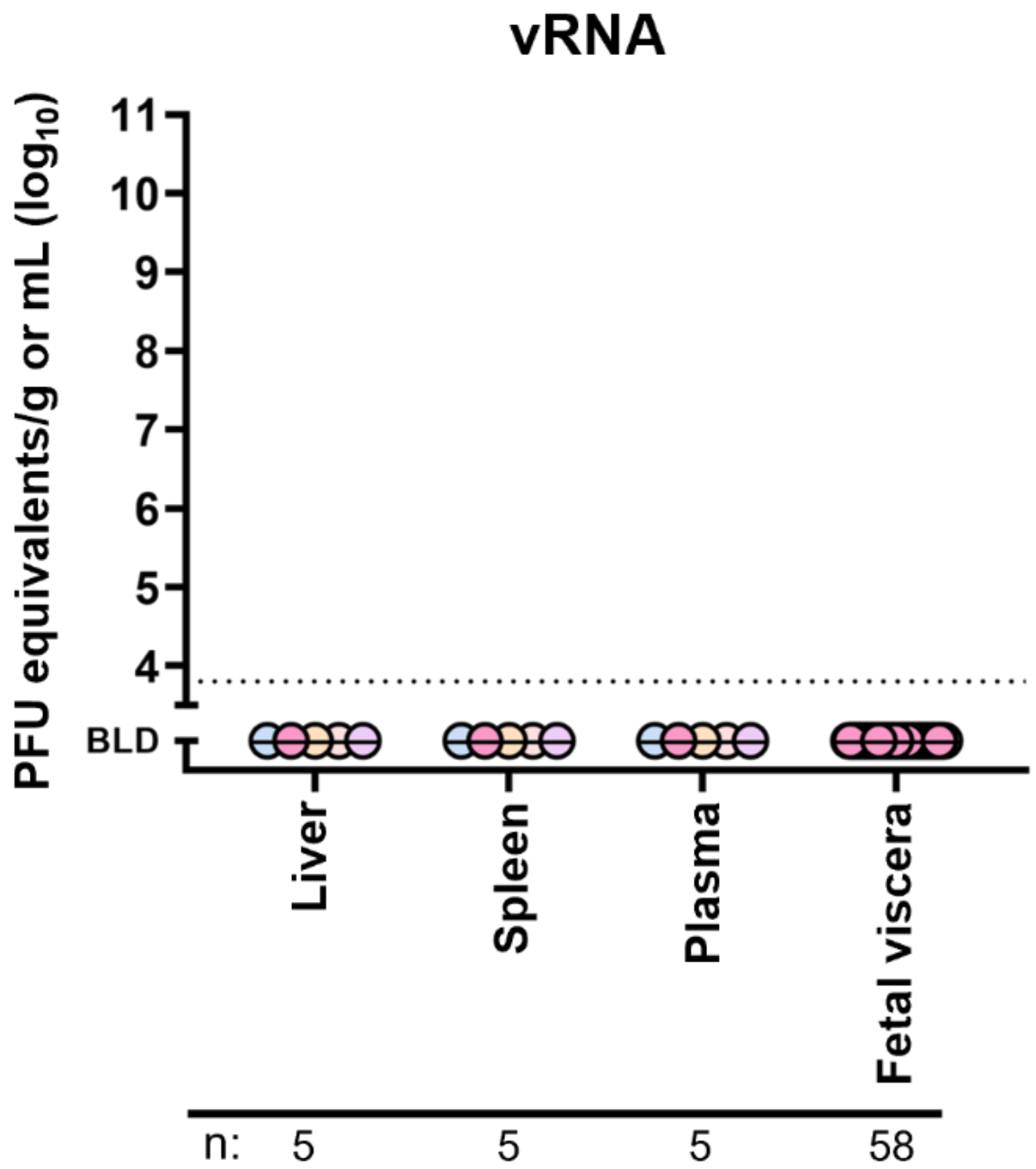

Fig S4. Dam:litter pairs vaccinated prior to pregnancy have no detectable vRNA following WT RVFV challenge. Viral RNA in maternal and pup samples at delivery (5-9 d.p.i.) following WT RVFV challenge. Each symbol color corresponds to individual dam:litter pairs. The dotted horizontal line represents the limit of detection of the q-RT-PCR assay (6400 PFU equivalents/g or mL). Data shown as geometric mean ± the geometric standard deviation.
